# Correction: Use of extracellular vesicles from lymphatic drainage as surrogate markers of melanoma progression and *BRAF*^*V600E*^ mutation

**DOI:** 10.1084/jem.2018152204162019c

**Published:** 2019-04-22

**Authors:** Susana García-Silva, Alberto Benito-Martín, Sara Sánchez-Redondo, Alberto Hernández-Barranco, Pilar Ximénez-Embún, Laura Nogués, Marina S. Mazariegos, Kay Brinkmann, Ana Amor López, Lisa Meyer, Carlos Rodríguez, Carmen García-Martín, Jasminka Boskovic, Rocío Letón, Cristina Montero, Mercedes Robledo, Laura Santambrogio, Mary Sue Brady, Anna Szumera-Ciećkiewicz, Iwona Kalinowska, Johan Skog, Mikkel Noerholm, Javier Muñoz, Pablo L. Ortiz-Romero, Yolanda Ruano, José L. Rodríguez-Peralto, Piotr Rutkowski, Héctor Peinado

Vol. 216, No. 5, May 6, 2019. 10.1084/jem.20181522.

The authors regret that in the original version of their paper, Laura Santambrogio and Mary Sue Brady were inadvertently omitted from the author list. The corrected author list appears above.

All versions of this article have been corrected.

